# Do poverty and wealth look the same the world over? A comparative study of 12 cities from five high-income countries using street images

**DOI:** 10.1140/epjds/s13688-023-00394-6

**Published:** 2023-06-07

**Authors:** Esra Suel, Emily Muller, James E. Bennett, Tony Blakely, Yvonne Doyle, John Lynch, Joreintje D. Mackenbach, Ariane Middel, Anja Mizdrak, Ricky Nathvani, Michael Brauer, Majid Ezzati

**Affiliations:** 1 Centre for Advanced Spatial Analysis (CASA), University College London, London, UK; 2 Department of Epidemiology and Biostatistics, Imperial College London, London, UK; 3 MRC Center for Environment and Health, Imperial College London, London, UK; 4 Melbourne School of Population and Global Health, University of Melbourne, Melbourne, Australia; 5 Public Health England, London, UK; 6 School of Public Health, University of Adelaide, Adelaide, Australia; 7 Bristol Medical School Population Health Sciences, University of Bristol, Bristol, UK; 8 Department of Epidemiology and Data Science, Amsterdam UMC–Vrije Universiteit, Amsterdam, The Netherlands; 9 School of Arts, Media and Engineering, Arizona State University, Tempe, USA; 10 Department of Public Health, University of Otago, Wellington, New Zealand; 11 School of Population and Public Health, The University of British Columbia, Vancouver, British Columbia, Canada; 12 School of Computing and Augmented Intelligence, Arizona State University, Tempe, USA; 13 Institute for Health Metrics and Evaluation, University of Washington, Seattle, WA USA; 14 Abdul Latif Jameel Institute for Disease and Emergency Analytics, Imperial College London, London, UK; 15 Regional Institute for Population Studies, University of Ghana, Accra, Ghana

**Keywords:** Street images, Visual similarity, Computer vision, Urban inequalities

## Abstract

Urbanization and inequalities are two of the major policy themes of our time, intersecting in large cities where social and economic inequalities are particularly pronounced. Large scale street-level images are a source of city-wide visual information and allow for comparative analyses of multiple cities. Computer vision methods based on deep learning applied to street images have been shown to successfully measure inequalities in socioeconomic and environmental features, yet existing work has been within specific geographies and have not looked at how visual environments compare across different cities and countries. In this study, we aim to apply existing methods to understand whether, and to what extent, poor and wealthy groups live in visually similar neighborhoods across cities and countries. We present novel insights on similarity of neighborhoods using street-level images and deep learning methods. We analyzed 7.2 million images from 12 cities in five high-income countries, home to more than 85 million people: Auckland (New Zealand), Sydney (Australia), Toronto and Vancouver (Canada), Atlanta, Boston, Chicago, Los Angeles, New York, San Francisco, and Washington D.C. (United States of America), and London (United Kingdom). Visual features associated with neighborhood disadvantage are more distinct and unique to each city than those associated with affluence. For example, from what is visible from street images, high density poor neighborhoods located near the city center (e.g., in London) are visually distinct from poor suburban neighborhoods characterized by lower density and lower accessibility (e.g., in Atlanta). This suggests that differences between two cities is also driven by historical factors, policies, and local geography. Our results also have implications for image-based measures of inequality in cities especially when trained on data from cities that are visually distinct from target cities. We showed that these are more prone to errors for disadvantaged areas especially when transferring across cities, suggesting more attention needs to be paid to improving methods for capturing heterogeneity in poor environment across cities around the world.

**Supplementary Information:**

The online version contains supplementary material available at 10.1140/epjds/s13688-023-00394-6.

## Introduction and background

Inequalities are on the rise globally and increasingly recognized as a social, political, and ethical problem [1–3]. Cities are fundamental in addressing inequalities as they account for 55% of the global population, and 81% in high-income countries where rich and poor are side by side (United Nations, 2019). The COVID-19 pandemic and recent extreme weather events have exposed spatial and environmental inequalities in cities and their impacts on health and wellbeing [4–6]. For example, poor and wealthy areas of a city can differ in access to green spaces and tree canopy, walking and cycling infrastructure, safety, density, overcrowding and quality of housing, and types of services and shops [7–12]. City governments have the tools to influence such features to help provide solutions to spatial and social inequalities. Many of these features are visible yet, there are no data on similarities or differences in the urban environments, and their visual representation, experienced by the wealthiest and poorest city dwellers around the globe. Such comparative understanding is crucial to cities globally to design effective interventions suited to local needs.

Visual information from streetscapes have been used to study urban inequalities at least as far back as Charles Booth’s ‘Inquiry into the Life and Labour of the People in London’ in the 1880s [13]. Urban ground-level visuals provide a rich perspective on the environment that people experience: they contain information on types, materials and condition of buildings; extent and safety of roads and sidewalks; types and density of vehicles, people, cyclists, shops, public spaces, and other amenities; green and blue space; and sources of pollution [7, 9, 14, 15]. Photography projects have also captured stark visual differences of rich and poor neighborhoods [16–18] and change associated with gentrification [9].

Booth and much of the early work on visual features of cities relied on in-person site visits which restricted the areas of the city that they could study. Large scale street-level images are a source of city-wide visual information and allow for comparative analyses of multiple cities [19]. Computer vision methods can infer socioeconomic and environmental features from a large number of street images [7, 9, 20–22]. Work to date in using images to infer socioeconomic measures has been within specific geographies and have not looked at how they compare across different cities and countries. In this study, we aim to understand whether poor and wealthy groups live in visually similar environments across cities and countries using deep learning methods applied to street-level images. A comparative understanding of streetscapes, and their inequalities, in cities around the world can also guide how we envision, develop, and design equitable, inclusive, and resilient cities. This is also crucial for evaluating whether image-based measurements using deep learning methods, that have become increasingly popular over the past few years [7, 9, 20–22], are biased when trained on data from cities that are visually distinct from target cities.

**Figure 1:**
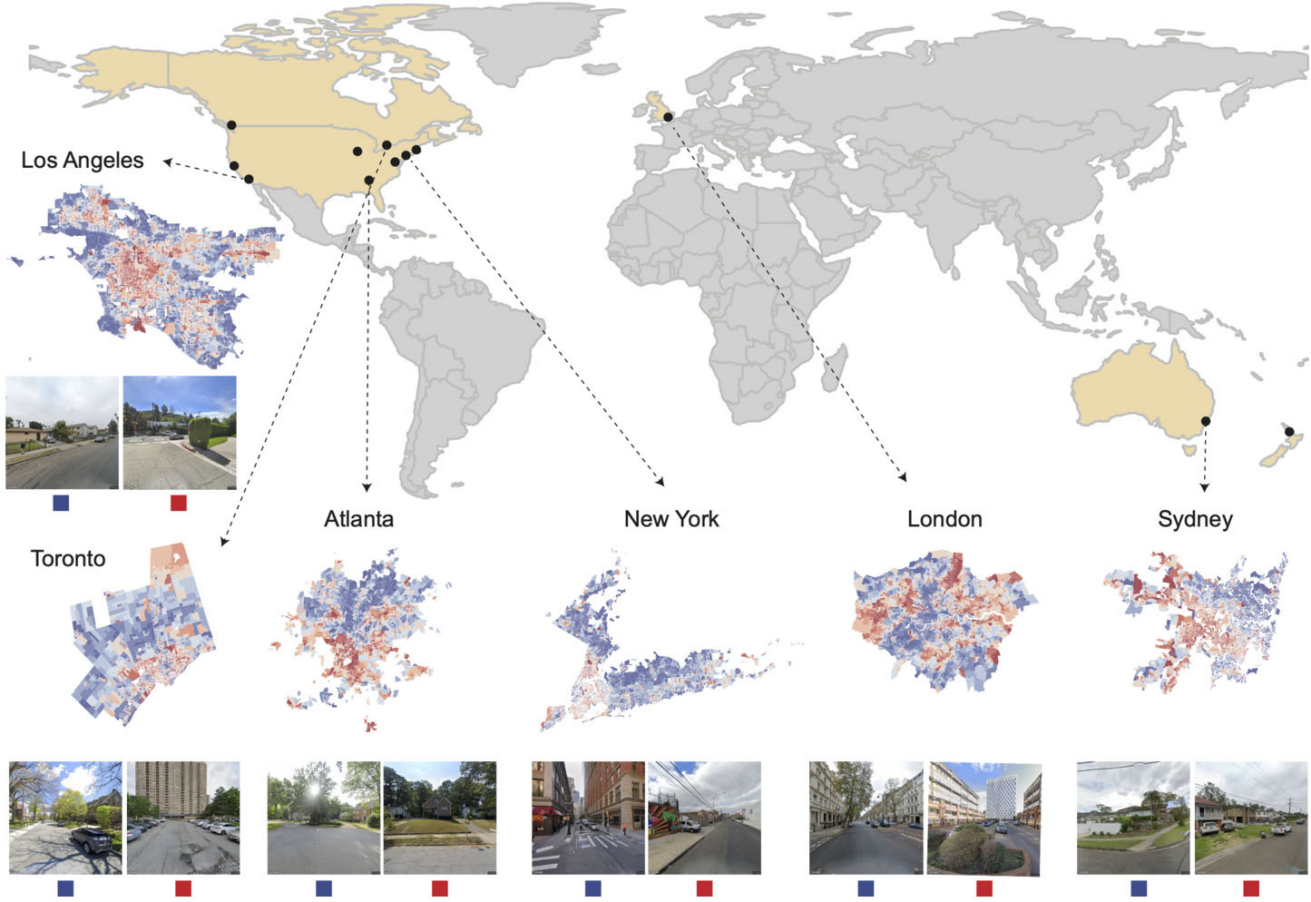
Overview of cities used in the analysis. Maps of deciles of income, with red indicating poorer areas and blue indicating more well-off areas, along with examples of street images are shown for selected cities. We used images from Google for our analyses; images shown here are courtesy of Mapillary

We studied similarities of visual features and distinctness of urban environments of the most and least advantaged communities across 12 cities in five countries, home to more than 85 million people: Auckland (New Zealand), Sydney (Australia), Toronto and Vancouver (Canada), Atlanta, Boston, Chicago, Los Angeles, New York, San Francisco, and Washington D.C. (United States of America), and London (United Kingdom) (Fig. 1). These cities are located in “English speaking” high-income countries where income inequalities are pronounced [23, 24]. We obtained images and data on household income at census tract level for all cities for comparative analyses. We divided census tracts into deciles of household income, with decile one corresponding to the worst-off 10% and decile ten to the best-off 10% of each city.

We analyzed a total of 7.2 million publicly available street images where we sampled a fixed number of street image locations for each census tract from Google Street View. Images were geotagged and assigned to the decile class of the corresponding census tract (See Materials and Methods for further details). We evaluated similarity in visual features between cities by measuring how well a model trained in one city performed when making income decile predictions using images from other cities [25]. We use the mean absolute error (MAE), defined based on the difference between predicted deciles and ground truth deciles, to measure the performance of the models.

We used a deep learning method to automatically recognize, i.e. learn, visual features from street images that are associated with different levels of income [7, 9, 20, 22, 26–31]. First, we trained our network using all image-label pairs from one ‘source’ city and the remaining cities were withheld. We then used the withheld images from ‘target’ cities to evaluate the transferability of the model. Mean absolute error (MAE) values were computed separately for each target city. We repeated this process 12 times using a different city as the ‘source’ each time so that we included all pairs of cities. In addition to inter-city comparisons, we tested the network within each city. For intracity performances, we trained networks separately for each city using five-fold cross validation. In each fold, image-output pairs for 80% of census tracts from one city were used for training our model and the remaining 20% were withheld. (samples stratified by deciles). Repeating this process five times holding out a different 20% of tracts each time, we computed the average MAE across the hold-out samples to evaluate intracity performance. We evaluated prediction performances for the best-off 10% and worst-off 10% tracts for each city, which represents extremes of inequality.

## Data and methods

### Definition of city boundaries and census tracts

Countries differ in how city boundaries are defined based on population size and density, as well as city history, urban function, and administrative and political organization. We used definitions by national statistical offices in each of the respective countries as follows (see Additional file 1 for detailed definitions). For Auckland, we used urban area (UA) boundaries as defined by Statistics New Zealand. For Sydney, we used significant urban area (SUA) boundary as defined by Australian Bureau of Statistics. For Toronto and Vancouver, we used census metropolitan area (CMA) boundaries as defined by Statistics Canada. For US cities in this study, we used urbanized area (UA) boundaries as defined by the US Census Bureau. For London, we used built-up area (BUA) (previously called urban areas) boundaries as defined by UK Office of National Statistics. Figure S2 shows each boundary with an underlying base map for each city.

For each of our study cities, we used the smallest standard geographic area i.e., census tract definition for which income data was available. We use Census Blocks for US, Lower Layer Super Output Areas for UK, Dissemination Areas for Canada, Statistical Areas Level 1 for Australia, and Statistical Area 1 for New Zealand (see Additional file 1 for detailed definitions). Average census tract population and the number of census tracts for each city are presented in Table S1.

### Image data

A total of 7,233,320 images from 15 cities were accessed using the Google Street View Application Programming Interface (API). We first created a 50 m grid for each of the cities using city boundary shape files. We then used the API to retrieve the unique panorama ids (‘panoid’) of images near each grid point acquired by Google for the years 2007-2019. A fixed number of panoids for each census tract were used prioritizing images taken in recent years. Census tracts are defined to have a similar population size for each country, yet their sizes vary across countries depending on how they are defined by statistical agencies in respective countries. We wanted our sampled number of images to be roughly proportional to the census tracts’ population. Therefore, we used 20 images from census tracts with smaller population sizes (i.e., Auckland, Sydney, Toronto, and Vancouver) and 30 images from census tracts from all other cities that have larger census tract populations (see Additional file 1, Table 1). We used 30 images per census tract for all other cities that have larger census tract populations. Census tracts within the city boundaries that did not have the required numbers of images, constituting 1% of all tracts, were excluded from our analysis. For each sampled panoid location, we used four images representing four orientations of a 360° panorama (0°, 90°, 180°, 270°) to estimate all directions within view. Table S1 shows the number of and average populations of census tracts for each city as well as the number of images obtained per census tract and in total.

### Data on income and education

Detailed data sources and metadata from national statistics offices for income and education for each of the cities are given in Additional file 1. For each outcome for each city, we calculated deciles of census tracts, with decile 1 corresponding to the worst-off 10% and decile 10 to the best-off 10%.

### Deep learning-based assignment to deciles

Following [22], we used pre-trained VGG16 [32] weights (trained on ImageNet [33] for object detection and classification i.e., not focusing on street images) as a fixed feature extractor to convert street view images to 4096 dimensional codes. We use the same network structure and loss function used by [22], where the inputs to the network are four images from one location and the output is an outcome decile (Figure S7). We trained for the weights of the fully connected layers using image-label pairs described in the previous section in all cities in our sample. The true labels used during training were the decile classes associated with the census tracts from which the images were sampled from. The network was trained using PyTorch in Python. For all experiments, we used the Adam optimizer with a learning rate 0.0001 and a batch size of 20 training the network for 30 epochs. The network that yielded the best validation error was kept as the final model.

### Measurement of prediction performance

For transferability performances, i.e., multicity experiments, all data from the source (i.e., training) city were used for training. We measured how well the trained network uses images to predict outcomes in all other target (i.e., prediction) cities that were not used in training [22]. We evaluated the performance using mean absolute error (MAE).

For evaluating how well models performed when making predictions for communities within the same city, i.e., single city experiments, we used five-fold cross validation. The census tracts of a city were randomly allocated to five equally sized groups of 20%. In each fold, 80% of census tracts (all images in these tracts) were used for training the network and the remaining 20% were withheld. We then measured how well the trained network uses images to predict outcomes for census tracts that were not used in training. We repeated this process five times for each of the cities holding out a different 20% of census tracts each time.

### Data on population density and distances to the city center

For a comparative measure of population density, we computed the number of people per km2 for each census tract. For distances to CBDs, we used coordinates for the CBD for each city from OpenStreetMaps and computed distances between the centroid point of each census tract and the CBD in kilometers.

Distributions of the wealthiest and poorest census tracts over space were substantially different for Canadian cities and most American cities compared to London, Sydney, and Auckland. Figure S5 and Figure S6 show comparisons in population density and distances to the CBD in each city between the highest and lowest income neighborhoods. North American cities are characterized by highest income groups living in car-dependent low-density suburbs whereas the lower income groups live near the center with high population density. The poor tend to live close to the city center as highlighted by the right-skewed distributions in Figure S5. Most of the highest income groups live farther away from the center as they have access to cars and ability to afford costs of private transport, and hence can afford to live farther from the center. Population density distributions (Figure S6) for high income groups are much narrower, which is intuitive as they have more opportunities to choose their neighborhoods to live in their preferred density. This is consistent with the suburbanization and car-oriented development history of American and Canadian cities, where high income people who can afford cars chose to move outside the city to consume more land [34] alongside the so-called “white flight” phenomenon ([35]).

These patterns, which will also have visual signs captured by street-level images, are reversed for London, Sydney, and Auckland where it is mostly the high-income groups that live closer to CBDs and the lower income groups father away from the center. Land near CBDs is valuable, and it is mostly the rich who can afford to live there as affordable housing in the center of these cities are scarce. The distributions of population density for the best-off 10% and worst-off 10%, as shown in Figure S6, are mostly overlapping where both income segments live in similar areas with respect to their density. In these cities, especially for London, dense, walkable, transit-oriented neighborhoods are also attractive for high-income segments of the population. San Francisco and New York had somewhat different patterns from other US cities, and similar to London, where instead many high-income neighborhoods are located near the center, populated by people who prefer to live near well-paying jobs and urban amenities.

### Regression analysis for predictors of similarity

We tested whether within city differences in population density and distance to city center as experienced by the wealthiest and poorest deciles would explain poorer transferability performance between city pairs. To do this, we needed a quantitative metric to summarize ‘the difference in differences’, comparing the difference in density and distance to CBD between the richest and poorest deciles for each city. We used the test-statistic of a two-sample t-test for each city and each metric; large positive or negative values of the test-statistic indicate strong evidence of a difference between richest and poorest deciles in that city, smaller values indicate weak or no evidence for a difference.

We then carried out a regression analysis (Eq. (1)) using the absolute difference between the test-statistics in the two cities for each of the metrics as covariates. We also included city-specific intercepts (constants) as well as an indicator variable for intra-country transfer. We ran separate estimations for the best-off and worst-off deciles (Table 1).

1 $$ \mathrm{MEA}_{st} = \mathrm{CSC}_{t} + \beta _{1} * I_{\mathrm{intra}} + \beta _{2} * \mathrm{ddensity}_{st} + \beta _{3} * \mathrm{ddistance}_{st}, $$

where *s* and *t* are source and target cities respectively. CSC is the city specific constant to be estimated, *β* are parameters to be estimated. ddensity is the difference between source and target city t-statistics computed for population density. ddistance is the difference between source and target city t-statistics computed for distance to CBD. $I_{\mathrm{intra}}$ is the indicator variable for intra-country transfer.

## Results

### Visual similarity between wealthiest areas compared to poorest areas

**Figure 2:**
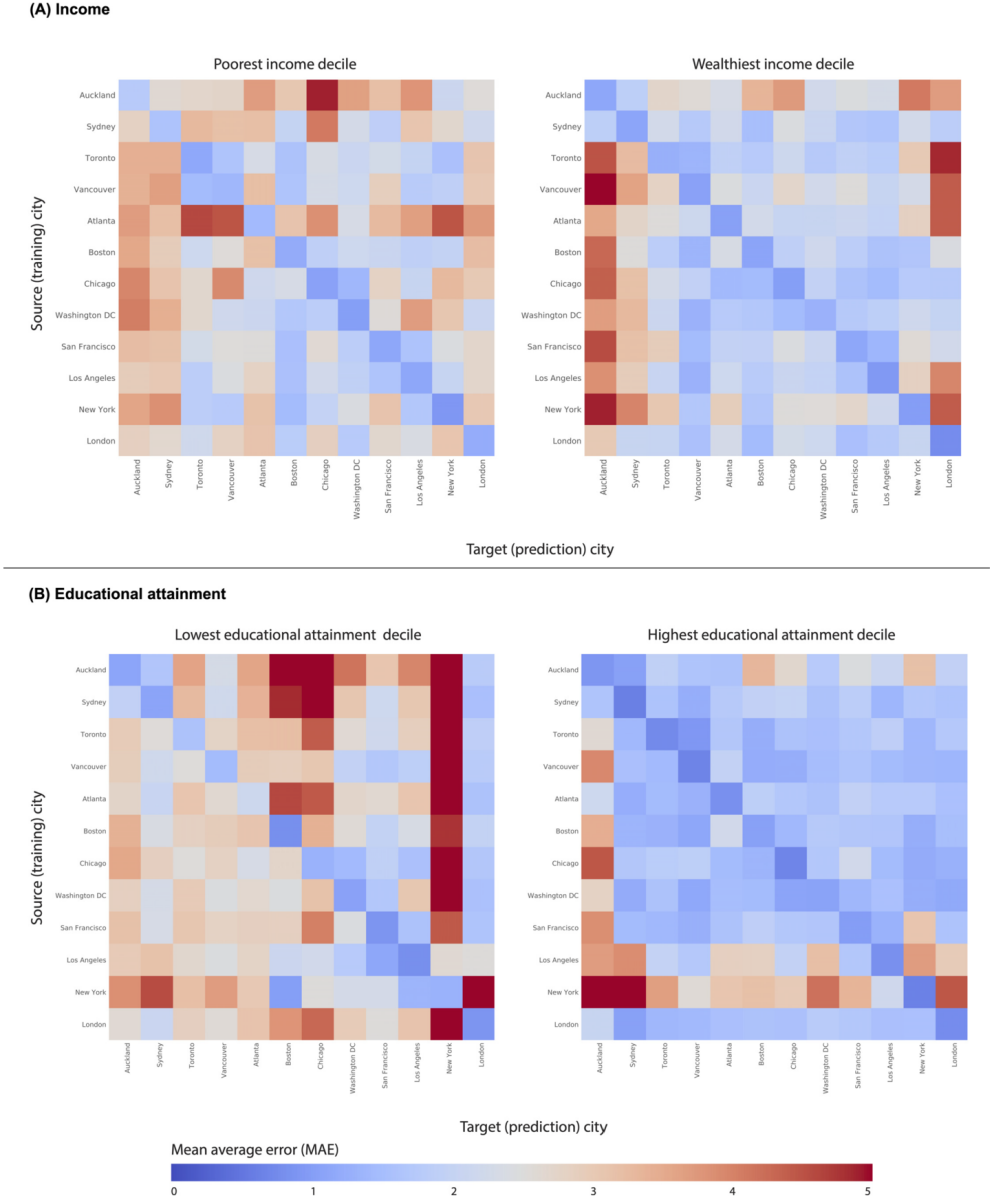
Measurement performances for all city pairs. Mean absolute errors (MAE) achieved when predicting worst-off and best-off deciles by (A) income and (B) educational attainment for all source (training) and target (prediction) city pairs. Diagonal entries show results using data from a single city as described in the text. Random allocation of census tracts to deciles in the target city would result in an MAE value close to 5 (dark red), whereas perfect prediction would correspond to a MAE value of 0 (dark blue). Shades of blue depict better prediction performances compared to shades of red

Tolstoy famously said “Happy families are all alike; every unhappy family is unhappy in its own way [36].” In the context of different neighborhoods in cities, higher-income residents have more opportunities to choose their neighborhoods to live in their favored neighborhoods aligned with their preferences across many dimensions (e.g., proximity to services, access to green space and parks, density, quality of infrastructure). Conversely, lower-income residents face significant trade-offs when deciding where to reside, often having to make do with what is available and affordable, which can vary greatly between cities. As a result of these influences, we hypothesized that well-off residents would live in visually similar neighborhoods, while deprived neighborhoods would be more heterogenous. Indeed, when we use street-level images, we found that well-off neighborhoods look relatively alike while deprived neighborhoods are more distinctive, especially in different cities using a similarity metric based on transferability performance of our deep learning model. Specifically, mean absolute error values were consistently lower when predicting the best-off 10% than predicting the worst-off 10% as shown in Fig. 2 and Table S2 (67% of transfer city pairs). This was consistent for within-city and across city transferability experiments, being more pronounced in the latter. There was a significant difference in across city MAEs for best-off (mean = 2.44, median = 2.19) and worst-off (mean = 2.69, median = 2.68) deciles using the t-test on two related samples of scores ($p < 0.001$). When we only look at within city comparisons using cross-validation, the MAEs of best-off (mean = 1.10, median = 1.10) were lower than MAEs of worst-off (mean = 1.26, median = 1.18) deciles, but this difference was not significant. Significant differences between cross-city MAEs suggest that visual features associated with poorer areas are distinct and unique to each city compared to wealthier areas that share more visual features across countries.

**Figure 3:**
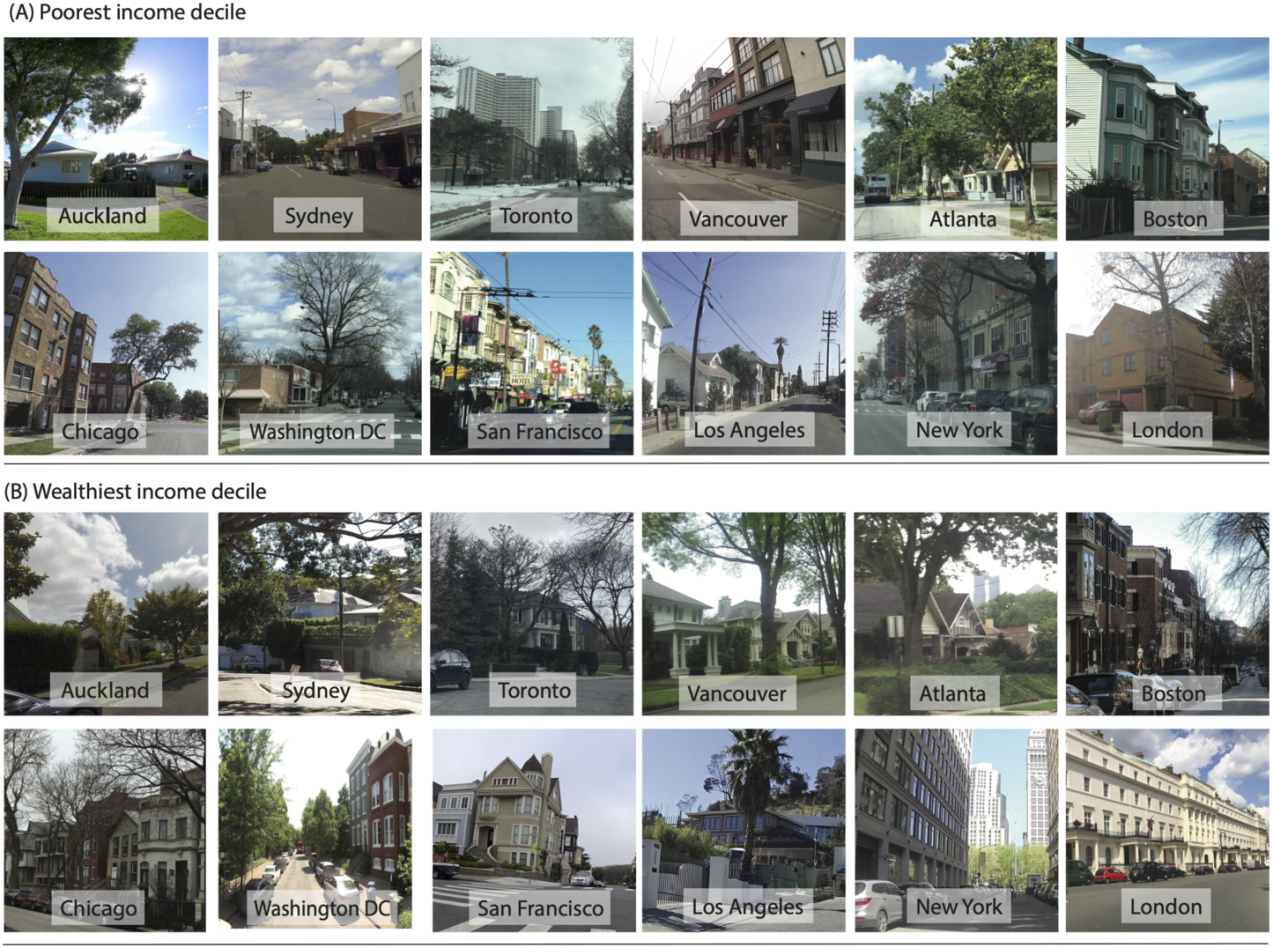
Example images. Street-level image examples from (A) poorest and (B) wealthiest communities in each city. Images courtesy of Mapillary

Poor areas in all cities display signs of disrepair and poor condition of pavements, roads, homes, and gardens, but differ in other features such as building design. As examples, Fig. 3 shows images from poorest and wealthiest communities in different cities. Images of poorly maintained social housing and tower blocks are symbols of overcrowding and poverty in London and Toronto (MAE of 2.22 and 3.10 where Toronto and London are target cities respectively), in contrast to Atlanta’s poor living in low-density suburbs (MAE of 4.6 and 3.72 for Toronto and London as target and Atlanta as source cities). Cars are the dominant mode of travel for all segments of population in cities such as Auckland and Atlanta with low population density, in contrast to cities like San Francisco and London where public transport is widely accessible. Trees and green space are visible in images of low-density neighborhoods of cities like Atlanta but are also of different character (e.g., large areas of greenspace containing gravel roads and nature paths) compared to urban parks and trees visible in higher density neighborhoods of London or New York (average MAE of 4.06 and 3.67 respectively for London and New York as target and Atlanta as source cities).

Wealthier residents can afford to live in neighborhoods with better amenities [8], as a result of which higher quality spaces look visually similar even in different countries. Another potential driver for higher similarity of higher-income neighborhoods may be related to there being a generic aspirational vision of what a well-off neighborhood looks like (e.g., new and well-maintained homes, low disorder) that may be in part shaped by media. There is nonetheless between-city heterogeneity, albeit to a lesser extent, in what constitutes a wealthy neighborhood. For example, wealthy communities where people live in apartment buildings in London and New York contrast to houses in most other American cities. Luxury apartment buildings in financial districts of New York and London with access to urban parks are visually distinct from the luxury mansions of Los Angeles with well-maintained private gardens (See Figure S4 for prediction maps for London when transferring from different target cities).

### Comparisons between cities within the same country and across different countries

Poor and rich communities looked more similar to their counterparts in other cities in the same country, than to those in cities in different countries. Models trained on data from an US city made better predictions in other US cities than in non-US cities, as was the case for Canadian cities. The same pattern was observed between Sydney and Auckland, which are in the pacific region although not in the same country. These cities share similarities in their climates, cultures, social and political history, and architecture. We also hypothesized that neighborhoods in big cities with populations of more than 5 million each (New York, Los Angeles, London, Chicago, Toronto, Sydney) would be more similar. However, we did not observe this from our results.

In addition, poor prediction performances for Auckland, Sydney, London, and Atlanta when transferring from other source cities (average MAE of 3.42, 3.10, 2.87 and 2.85 respectively as target cities) are consistent with observed differences in spatial patterns described in Sect. 2.7. It mirrors local differences, for example, that the well-off residents of London live in areas characterized by high building density and are hence visually distinct from the well-off suburbs in Los Angeles.

### Predictors of similarity

**Figure 4:**
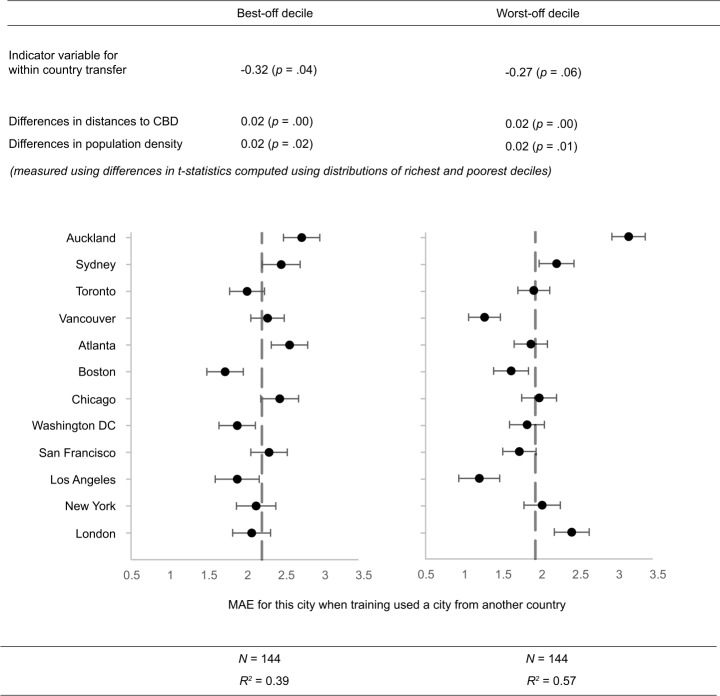
Multivariable regression of prediction errors (MAE). Between city MAEs were regressed against an indicator for whether the predicted and training cities were in the same country, differences in population density and distances to city center. Separate regressions were carried out for the best-off and worst-off deciles. Coefficients of ‘differences in distances to CBD’ and ‘differences in population density’ show change in error for each unit of difference in t-values. (See Materials and Methods for further details of variables included and how the difference in t-values were computed)

We considered differences across cities between the highest and lowest income deciles in two well-defined and quantifiable features described above: their population density and distances to the city’s central business district (CBD), as predictors of inter-city similarity. Both features have direct visual signs, such as more buildings, cars, and less sky view, that are captured by street images. To summarize and quantify the role of differences and similarities observed in Figure S5 and Figure S6, we conducted regression analyses (Fig. 4). Indeed, we find that the magnitude and direction of differences in population density and distance to city center between poor and wealthy neighborhoods partially explain their visual similarities between cities.

Between city MAEs were regressed against an indicator for whether the predicted and training cities were in the same country, and differences in population density and distances to city center between the corresponding two cities (Eq. (1)). The test-statistic corresponds to a two-sample t-test where large positive or negative values of the test-statistic indicate strong evidence of a difference in the metric (e.g., population density) between tracts in lowest and highest income deciles within the same city, and smaller values indicate weak or no evidence for a difference. The absolute difference between the test statistics computed for two cities were then used as the co-variate in the regression model as a measure of relative difference in distribution of each metric across these two cities. The differences in t-statistics computed between two cities ranged between 0.02 and 44.89.

Regression results revealed that a moderate share of variation in transferability errors ($R^{2} = 0.39$ for best-off and $R^{2} = 0.57$ for worst-off deciles) is explained by variables included as shown in Fig. 4 including city specific intercepts. The intra-country transfer indicators coefficients were negative as it is harder to transfer across countries than within country. It showed that, compared to within-country transfer, cross country would have 0.32 and 0.27 more error on average (p-values = 0.04 and 0.06, respectively) for allocation to the best-off and worst-off deciles. Coefficients for differences in population density and distances to the center between rich and poor communities were positive: the bigger the difference between source and target cities, the harder it is to transfer. Observed distributions of population density and distance to CBDs are related to how cities developed over time, physically and administratively, including decentralization patterns and car-oriented development.

### Comparison with similarities of communities based on educational attainment

Correlations between educational attainment and household income (both measured at the census tract level) varied across cities ranging from r = 0.17 for Vancouver to r = 0.81 for London and Atlanta (Figure S3). In Canada, many highly educated immigrants cannot get high paying jobs as their credentials are not recognized (Picot et al., 2008). Similar yet less pronounced dynamics are in play for both Sydney and Auckland. When we repeated the same set of analyses with street images using educational attainment instead of income, we found even higher visual similarity between areas with highest educational attainment. Lowest educational attainment tracts were even more different from each other (Fig. 2). For 80% of transfer city pairs, MAE were lower when predicting the best-off 10% than predicting the worst-off 10%. Differences between MAEs of best-off (mean = 1.90) and worst-off (mean = 2.83) deciles ($p < 0.001$) were larger.

Greater visual similarity between highest education neighborhoods is consistent with evidence that amenity levels (which will have stronger visual signals) have more of an influence on high-skill workers’ decisions of where they live compared to low-income workers where the priority is on rents and wages [37, 38]. Transferability performances were particularly poor for predicting lowest education decile areas in New York. Higher dissimilarity between lowest education neighborhoods is consistent with evidence of higher segregation of high- and low-skill workers into different cities. Certain larger cities by population (e.g., New York), for instance, attract both higher- and lower-skilled workers increasing inequalities whereas smaller cities have thinner tails in skill distribution [39] potentially leading to distinct visual features we observe in our work.

## Discussion and limitations

Plato’s statement from more than 2300 years ago, that “any city, however small, is in fact divided into two, one the city of the poor, the other of the rich,” still holds true today as neighborhood experiences of the wealthiest and poorest residents differ widely even when they live in the same city [40, 41]. To probe the characteristics of this feature in multiple cities, we asked: do the environments of the rich (and the poor) look similar in different cities across the world? Using transferable models, we showed that visual indicators of wealth across cities are more similar than those of poverty.

In previous work, visual elements in street images, including window and roof styles, number and types of cars, store signs, trees, fire escapes, and road widths, were found to be correlated with socioeconomic variables in city or country specific studies [7, 9, 20, 21]. Parallel to this, computer vision methods can also identify distinct architectural elements for different cities (e.g. lampposts, doors, balconies, windows with railings) [42]. Taken together, this body of work shows that poverty and affluence in different cities have distinct as well as related features. We did not extract pre-defined features so that we are not limited by the type of visual features we can extract i.e., for which sufficient labeled datasets or pre-trained models exist. Instead, we let the model learn visual features associated with wealth or poverty in a city, and test whether it predicts the same social phenomenon elsewhere. It enables the networks to make use of all features visible from images that potentially impact people including but not limited to types and conditions of buildings, vehicles, people, shops, pedestrian and cycling infrastructure, green and blue space, aesthetics, and trash on streets. This approach is akin to how humans intuitively identify where a poor or wealthy neighborhood may be, even in a city that they have not been to before. The limitation with this approach, however, is that post-hoc analysis is needed to identify which visual features, that are ideally policy amenable, are driving the differences.

We find that, only some visual elements associated with poverty or wealth in one city only generalize to others. Similarities as well as differences across cities are driven by historical factors, policies, and local geography. Car-oriented development often results in lower density especially when geography allows for city expansion. Poor suburban neighborhoods often have green space visible from images. However, in such cities, poor neighborhoods often lack access to sufficient levels of urban amenities, affordable and sustainable transport options. On the other hand, cities that have higher shares of public transit use are often higher in density. From what is visible from street-level images, high-density poor neighborhoods lack trees or green space in proximity. In addition, residents in such neighborhoods may be more exposed to hazards related to traffic such as higher levels of pollution, and road collisions. They may also suffer from poorer municipal services including waste collection. Further, residents of such neighborhoods may enjoy higher levels of access to urban amenities, but the type and quality of amenities may be poor and inadequate for the population density (i.e., overcrowding). While we used the full dataset containing all deciles from all cities during training, we used the best-off and worst-off deciles for our evaluations relating to the wealthiest and poorest areas in each city in line with the focus of the paper. Similarities or differences in other deciles and their transferability across cities and countries, which are arguably harder to interpret, are not part of our analysis.

Cities are dynamic and their visuals change with poor and rich residents moving in and out of neighborhoods, city investments and regulations. History of migration and gentrification also affect visual similarities, where new social groups settle into existing neighborhoods changing their social fabric and environmental quality. For example, land ownership changes over time where the wealthy migrate according to changing opportunities (e.g., new developments near particularly attractive areas new waterfronts), sometimes pushing out the poor [43]. Features like cars, buildings, shops, and aesthetics also change over time. Such dynamics are not captured in our cross-sectional analysis yet are visible from time-varying street images that are increasingly available.

Methodologically, we found that transferability errors were higher for poverty compared to that of wealth. This finding raises the need for caution in using imagery for measurements of socioeconomic status for data-poor geographies [44]. Specifically, greater error when predicting deprived areas can have serious implications. For example, if used for resource allocation, there has to be effort in additional data to understand the real nexus of where the poor are.

In high-income countries, inequalities are increasingly concentrated in urban areas [6, 45]. While city governments have limited influence on national economic policies, they can influence and change cities’ built environment to benefit disadvantaged residents. Examples of feasible actions include allocation of land for parks and nature, public spaces, car free zones, higher quality homes, improving foodscapes, civic spaces for all age groups, improving public transit networks and stops including public transit only lanes, better infrastructure for walking and cycling. The focus on ‘building back better’ and post-pandemic cities offers the chance to remedy our cities. Data-driven empirical findings enabled by our work and methods will support generation of novel and strategically relevant hypotheses to help identify urban policy and planning actions.

## Supplementary Information

Below is the link to the electronic supplementary material.

Supplementary information (PDF 8.0 MB)

## Data Availability

All datasets used in this paper are publicly available and sources are provided in the main manuscript and Additional file 1 section. Upon publication, the code will be available at.
