# Supplementary material for: Do poverty and wealth look the same the world over? A comparative study of 12 cities from five high-income countries using street images
Source: EPJ Data Sci. 2023 Jun 7;12(1):19. doi: 10.1140/epjds/s13688-023-00394-6 (PMC10245348; doi:10.1140/epjds/s13688-023-00394-6)
Supplement: Supplementary file 1 — Supplementary information (PDF 8.0 MB) [file 13688_2023_394_MOESM1_ESM.pdf]

# Supplementary Information

**Do poverty and wealth look the same the world over? A comparative study of 12 cities from five high-income countries using street images.**

**This PDF file includes:**

Supplementary Text
Figures. S1 to S7
Tables S1 to S2

**Supplementary Text**

1. Study Area

**Definition of city boundaries.** City boundaries was defined based on definitions used by national statistical offices in each country. Figure S2. shows the extent of each boundary with an underlying base map for each city. We used the following definitions in this study:

- For Auckland in New Zealand, we used urban area (UA) boundaries as defined by Statistics New Zealand. These areas are statistically defined with population of 1,000 or more with no administrative or legal basis.
- For Sydney in Australia, we used significant urban area (SUA) boundary as defined by Australian Bureau of Statistics. These are areas of concentrated urban development with a population over 10,000 representing one or more urban centers.
- For Vancouver and Toronto in Canada, we used census metropolitan area (CMA) boundaries as defined by Statistics Canada. These are areas consisting of one or more neighboring municipalities situated around a major urban area with a population over 100,000 of which a minimum 50,000 living in the urban core.
- For Atlanta, Boston, Chicago, Los Angeles, New York, San Francisco, and Washington DC in the US, we used urbanized area (UA) boundaries as defined by the US Census Bureau. These are urban areas with a population over 50,000 and may serve as the core of a metropolitan statistical area (MSA).
- For London, we use built-up area (BUA) (previously called urban areas) boundaries as defined by UK Office of National Statistics. These areas are defined as land that are irreversible urban in character.

**Definition of census tracts.** For each of our study cities, we used the following smallest standard geographic area i.e., census tract definition for which income and education data was available at. Average census tract population and the number of census tracts for each city are presented in Table S1.

- For Auckland in New Zealand, we used Statistical Area 1 (SA1) consisting of 100-500 persons.
- For Sydney in Australia, we used Statistical Areas Level 1 (SA1) consisting of 200-800 persons.
- For Vancouver and Toronto in Canada, we used Dissemination Areas (DAs) consisting of 400-700 persons.
- For Atlanta, Boston, Chicago, Los Angeles, New York, San Francisco, and Washington DC in the US, we used Census Block Groups consisting of 600 to 3000 persons.

- For London in the UK, we used Lower Layer Super Output Areas (LSOAs) consisting of 1000 to 3000 persons.

2. Income and education data

**Income data.** All study cities had information on household income at the small area level. Income information is available using national currencies, and there are differences in their measurement and reporting. Figure S1 shows the distribution of absolute values using 2020 inflation adjusted US dollars. Note that these values are not directly comparable due to differences in how they are being computed with respect to the reporting of mean vs. median values as well as the use of group brackets for some cities. For our study we used relative, and not absolute, values. We computed deciles of income based on the variables detailed below, where decile 1 corresponded to the bottom (poorest) 10% of census tracts and 10 to the top (richest) 10% of census tracts. Detailed for each city are as follows:

- For Auckland, we used median annual household income available from the 2018 Census reported by Statistics New Zealand.
- For Sydney, we used annual household income available from the 2016 Census reported by the Australian Bureau of Statistics. Number of households for each income bracket is reported. We computed the weighted average income value for each SA1 and used it to compute the deciles. The income group brackets used for reporting were as follows: Negative income; Nil income; $1-$10,399; $10,400-$15,599; $15,600-$20,799; $20,800-$31,199; $31,200-$41,599; $41,600-$51,999; $52,000-$64,999; $65,000-$77,999; $78,000-$103,999; $104,000 or more. We used midpoints of each interval, and the lower bound for the highest income group for computing the weighted average.
- For Toronto and Vancouver, we used annual median household income for each dissemination area for Toronto and Vancouver available from 2016 Census reported by Statistics Canada.
- For Atlanta, Boston, Chicago, Los Angeles, New York, San Francisco, and Washington DC, we used annual household income estimates available from the 2015-2019 American Community Survey (ACS) 5-year estimates. Number of households for income brackets are reported (variables B19001_001E to B19001_017E). We computed the weighted average income value for each census block group by multiplying counts (i.e., number of households reported for each interval). The income group brackets used for reporting incomes were as follows: Less than $10,000; $10,000-$14,999; $15,000-$19,999; $20,000-$24,999; $25,000-$29,999; $30,000-$34,999; $35,000-$39,999; $40,000-$44,999; $45,000-$49,999; $50,000-$59,999; $60,000-$74,999; $75,000-$99,999; $100,000-$124,999; $125,000-$149,999; $150,000-$199,999; $200,000 or more. We used midpoints of each interval, and the lower bound for the highest income group. The variable on median household income (B19013_001E) contained more missing values, hence we use the weighted average as explained here for computing income deciles.
- For London, we used Greater London Authority (GLA) mean annual household income estimates available for the year 2012/13. Income estimates take no account of average

household size or composition within each area. Income information is not available at small area level of other cities in the UK.

**Education data.** Each country reports information on educational attainment at the small area level. Due to differences in education systems and data reporting, there are some inconsistencies in definitions. We used the three following measures using equivalent degrees in each of the countries. $EDU_A$ is the one measure that is often used for measuring deprivation in educational attainment. However, most of our study cities did not show sufficient variation in $EDU_A$ and it was not possible to compute 10 deciles based on this variable only. Therefore, we also computed $EDU_B$ and used their average ($EDU_{score}$) as below for computing deciles used for educational attainment. We computed deciles of educational attainment, where decile 1 corresponded to the bottom 10% of census tracts and 10 to the top 10% of census tracts. Below we also summarize the variables used for computing both $EDU_A$ and $EDU_B$. Figure S1 shows distributions or each variable for each of the cities.

$$EDU_A = \text{Share of adults with a high school diploma or higher}$$
$$EDU_B = \text{Share of adults with a bachelor's degree or higher}$$
$$EDU_{score} = (EDU_A + EDU_B)/2$$

- For Auckland, educational attainment is reported at the individual level. We used the following from the 2018 Census reported by Statistics New Zealand: 'Share of People Aged 15 Years & Older with a High School Diploma or Higher' includes Level 3 (level3), Level 4 (level4), Level 5 (level5), Level 6 (level6), Bachelor's degree (level7_bs), Masters (masters), PhD (phd). 'Share of People Aged 15 Years & with a Bachelor's Degree or Higher' includes Bachelor's degree (level7_bs), Masters (masters), PhD (phd).
- For Sydney, educational attainment is reported at the individual level. We used the following from the 2016 Census reported by the Australian Bureau of Statistics: 'Share of People Aged 15 Years & Older with a High School Diploma or Higher' includes Secondary Year 12 (secondary_year12), Certificate 3-4 (certificate_3_4), Advanced (advanced), Bachelor's degree (bachelor), Graduate degree (graduate), Postgraduate degree (postgraduate). 'Share of People Aged 15 Years & with a Bachelor's Degree or Higher' includes Bachelor's degree (bachelor), Graduate degree (graduate), Postgraduate degree (postgraduate)
- For Toronto and Vancouver, educational attainment data is reported at household level based on a 25% sample data. We used the following from the 2016 Census reported by Statistics Canada: 'Share of households where the highest diploma or degree for adults Aged 25 to 64 Years is High School Diploma or Higher' includes Secondary (high) school diploma or equivalency certificate (Line 1700) and Postsecondary certificate, diploma or degree (Line 1701). 'Share of households where the highest diploma or degree for adults Aged 25 to 64 Years with a Bachelor's Degree or Higher' includes University certificate, diploma or degree at bachelor level or above (Line 1707)

- For Atlanta, Boston, Chicago, Los Angeles, New York, San Francisco, and Washington DC, we used educational attainment data from 2015-2019 ACS 5-year estimates. 'Share of Adults Aged 25 Years & Older with a High School Diploma or Higher' includes Regular high school diploma (B15003_017E), GED or alternative credential (B15003_018E), Bachelor's degree (B15003_022E), Master's degree (B15003_023E), Professional school degree (B15003_024E), Doctorate degree (B15003_025E). 'Share of Adults Aged 25 Years & Older with a Bachelor's Degree or Higher' includes Bachelor's degree (B15003_022E), Master's degree (B15003_023E), Professional school degree (B15003_024E), Doctorate degree (B15003_025E).
- For London, we used the following from the 2011 Census. 'Share of Persons Aged 25 Years & with a Bachelor's Degree or Higher' includes Qualification; highest level of : Level 4 qualifications and above for age groups above 25 (F316756, F358347, F358355, F316784), Share of Persons Aged 25 Years & Older with a Bachelor's Degree or Higher includes Qualification; highest level of : Level 3 qualifications for all age groups above 35 (F316755, F358346, F358354, F316783).

**Population density.** All study cities had information on number of persons living in each tract from the datasets used for obtaining outcome data on income and educational attainment as described above. For a comparative measure of population density, we compute the area in $km^2$ of each census tract from shapefiles. We then compute the population density as person per $km^2$ by dividing the number of persons living in that tract by its area using the latest Census available as detailed above.

**Distance to central business districts.** We used coordinates for the central business district (CBD) for each city available from OpenStreetMaps. The centroid point of each census tract was used to compute the Euclidean distances in kilometers of each tract to the CBD. The coordinates of CBDs used for each city are as follows: Auckland (-36.847, 174.765), Sydney (-33.868056, 151.212222), Toronto (43.652503, -79.383558), Vancouver (49.281762, -123.117718), Atlanta (33.755, -84.39), Boston (42.35, -71.066667), Chicago (41.881944, -87.627778), DC (38.9025, -77.032861), San Francisco (37.7952, -122.4029), New York (40.754931, -73.98402), Los Angeles (34.05, -118.25), and London (51.515556, -0.093056).

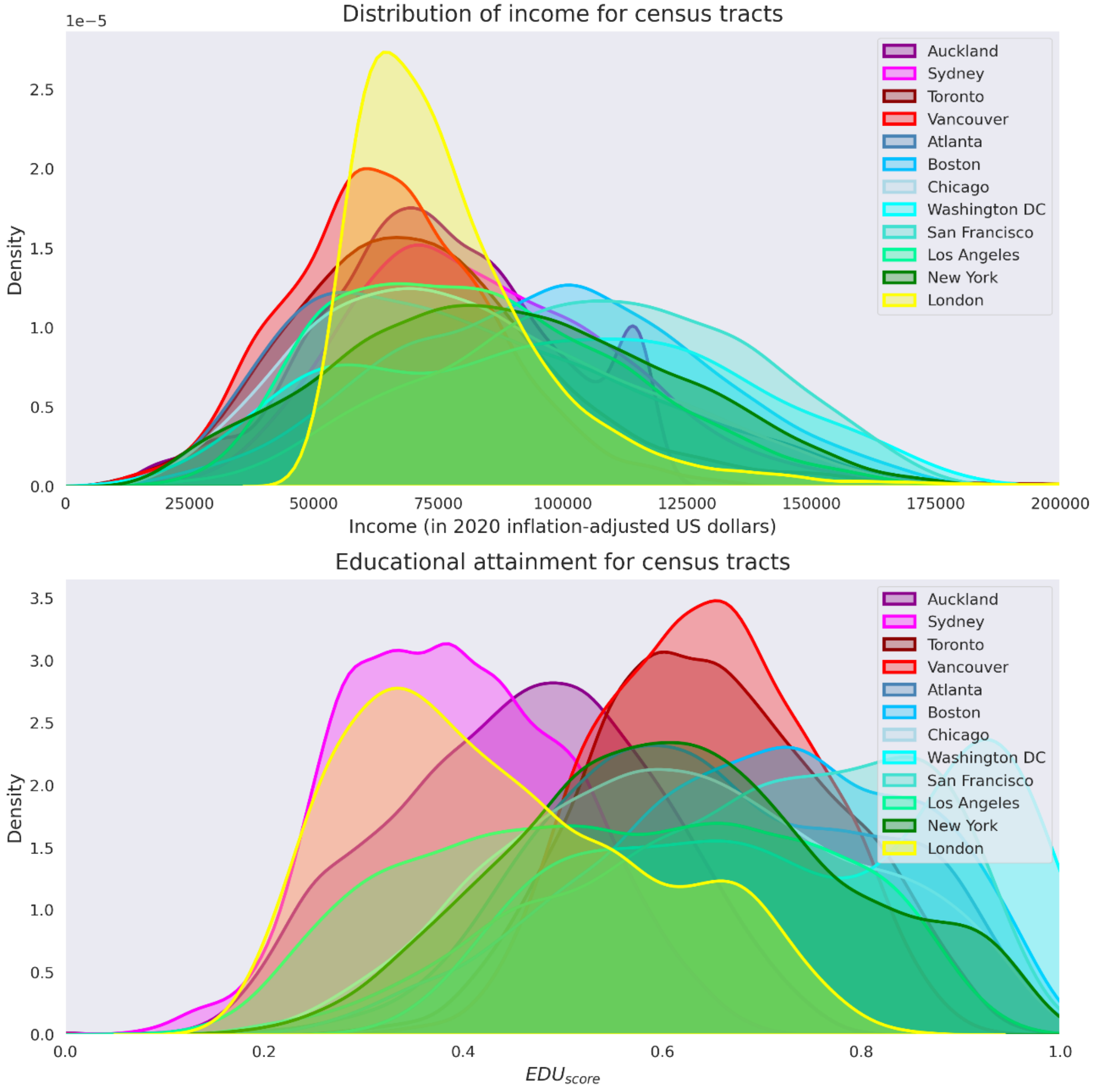


**Figure**

**Figure S1.** Distribution of income and education attainment at census tract level. Income is reported at the household level and was converted to 2020 inflation-adjusted US dollars for this figure. The bimodal distribution for Auckland results from how household income is reported where highest income groups (with annual incomes over NZ$150,000) are binned together.

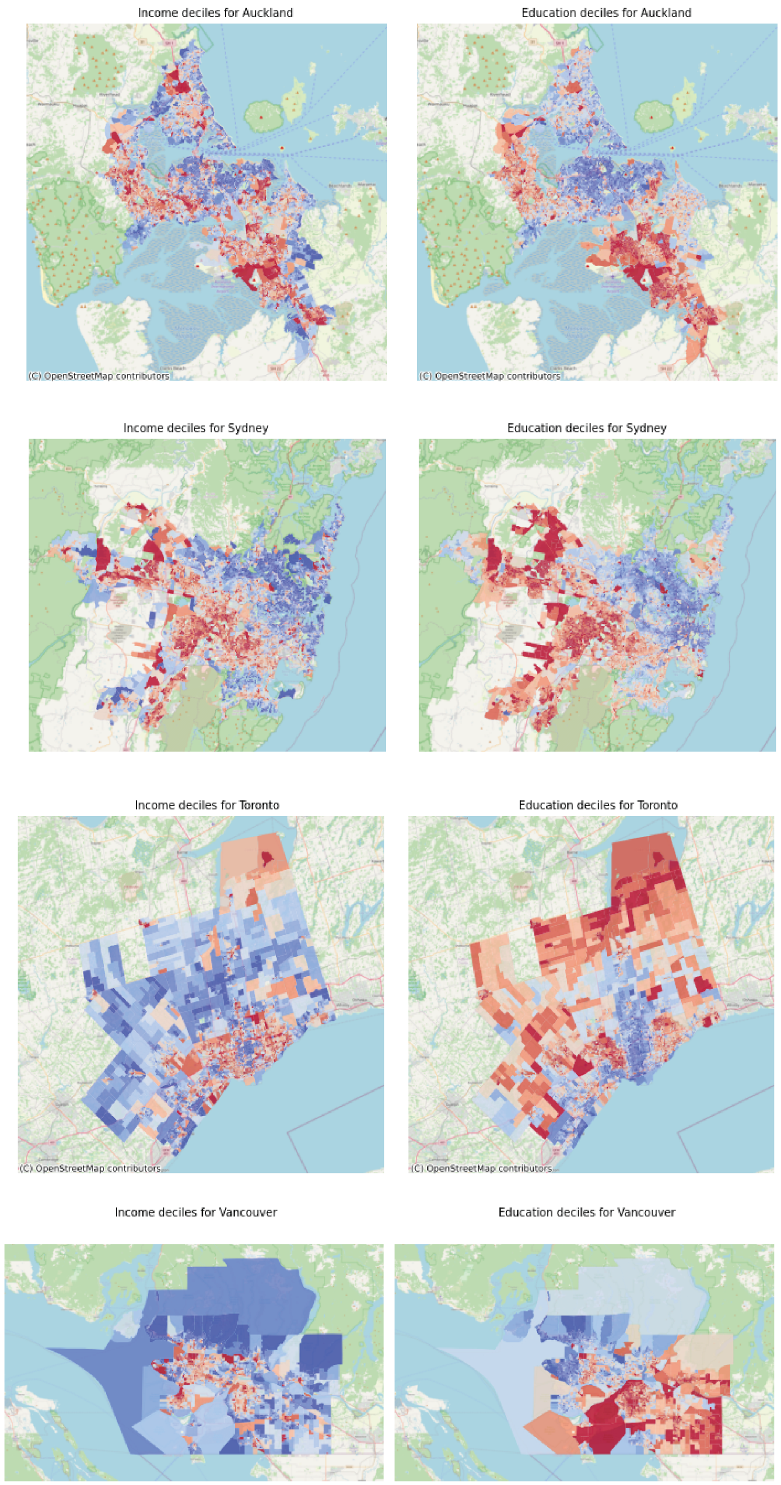


**Figure S2.A.** Income and education deciles within the city boundaries for Auckland, Sydney, Toronto, and Vancouver. Darker red colors correspond to the lowest (worst-off) deciles and darker blue colors correspond to highest (best-off) deciles both for income and education.

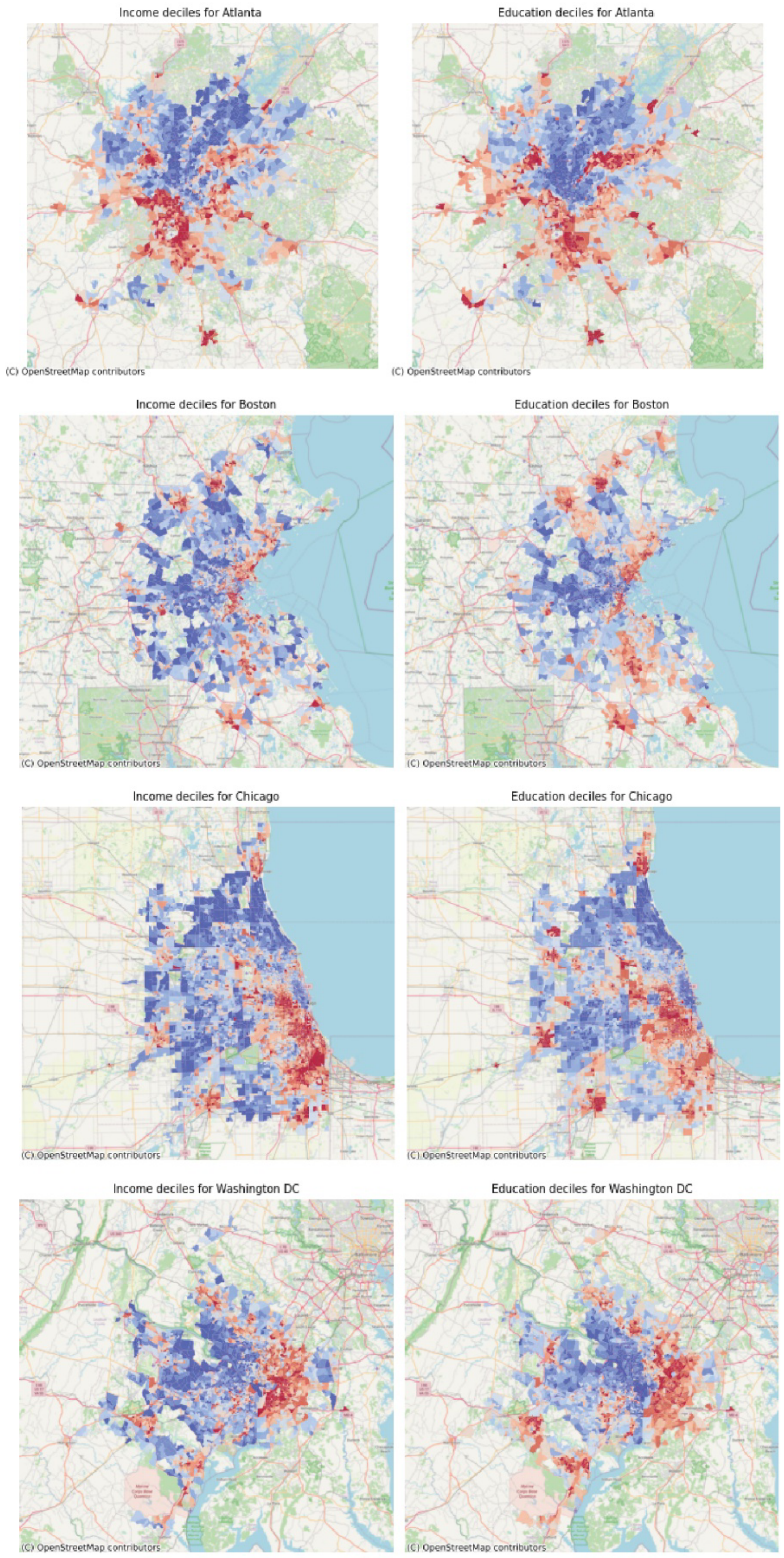


**Figure S2.B.** Income and education deciles within the city boundaries for Atlanta, Boston, Chicago, and Washington DC.

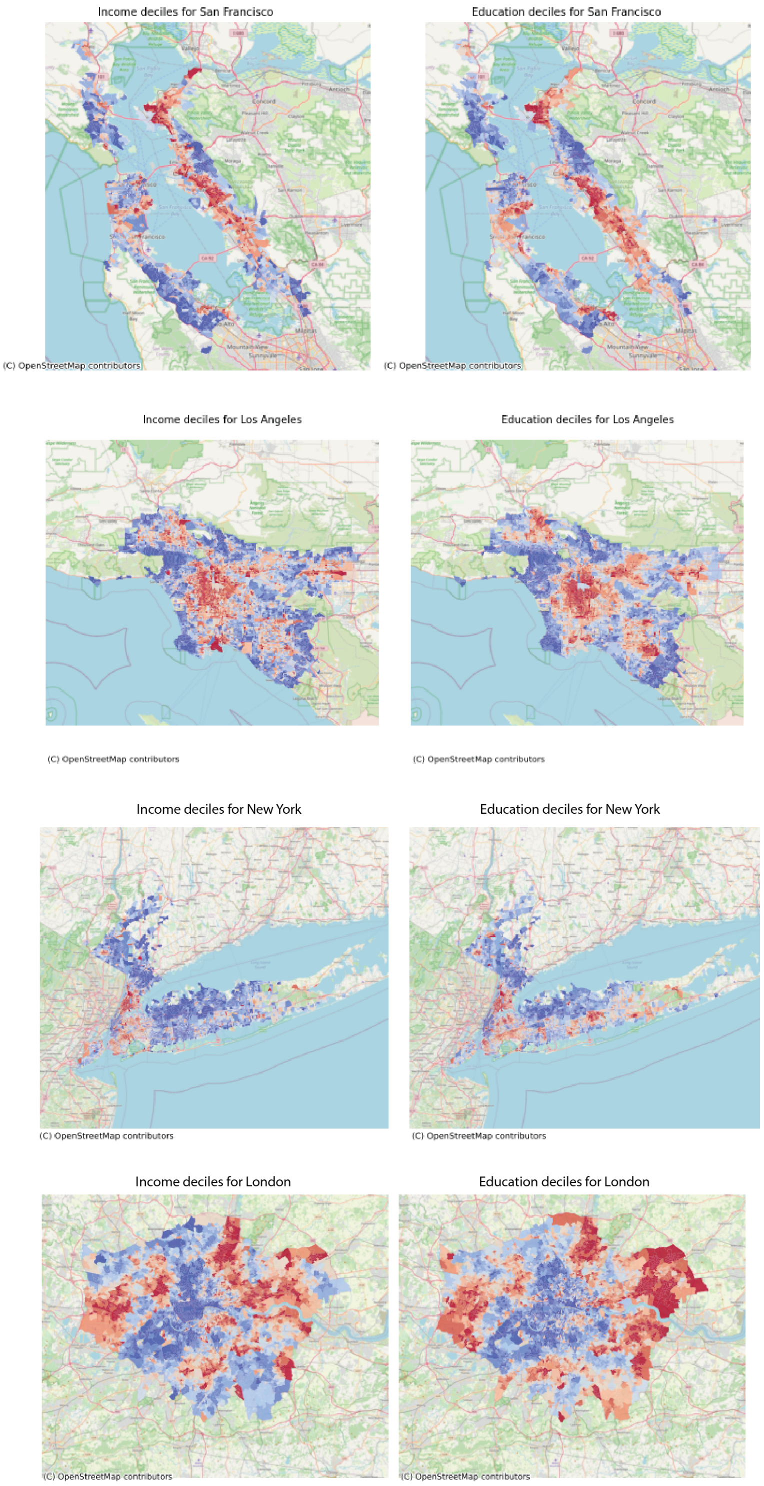


**Figure S2.C.** Income and education deciles within the city boundaries for San Francisco, Los Angeles, New York, and London.

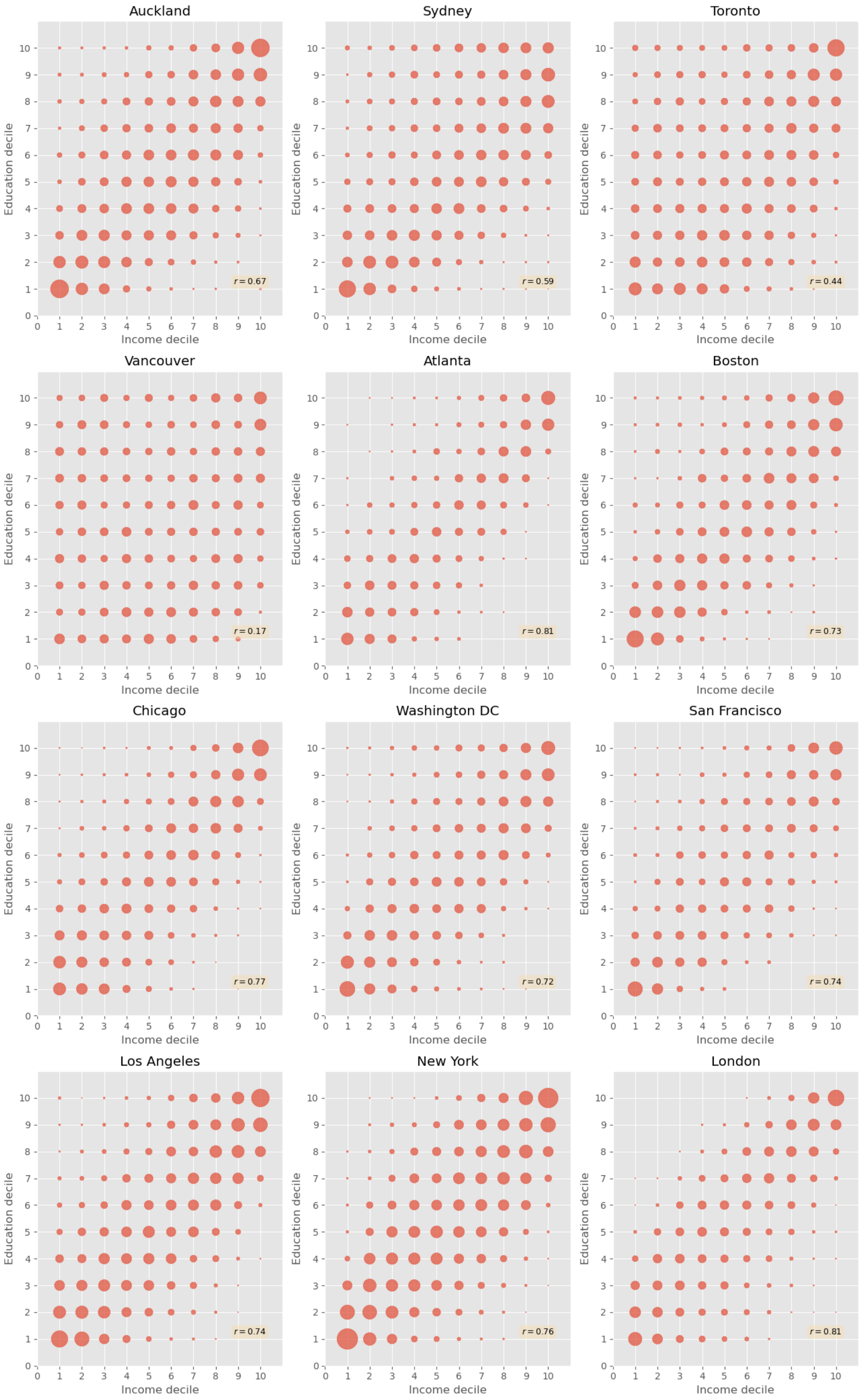


**Figure S3.** Concordance of income and educational attainment deciles ranging from relatively low ($r = 0.17$ for Vancouver and $r = 0.44$ for Toronto) to high ($r = 0.81$ for London and Washington DC).

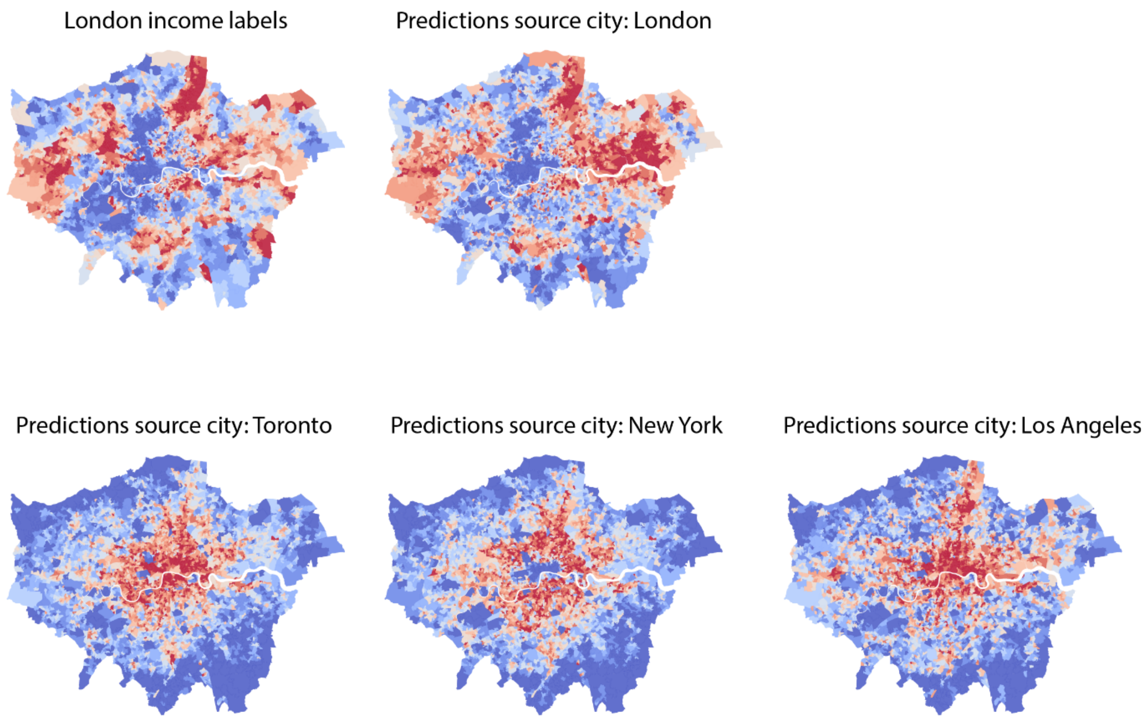


**Figure S4.** Label and income prediction maps for London. Darker red colors correspond to poorest deciles and the darker blue colors correspond to wealthiest deciles. Intracity model (when training and testing on London data) is successful in capturing the spatial variation across space. When the aim is to transfer from other cities (when training is done on another city's data and testing on London), however, spatial variation is not sufficiently captured. As examples, prediction maps from Toronto, New York, and Los Angeles reveals the differences in urbanization patterns in these cities. Specifically, Toronto's poor mostly lives in very high-density neighborhoods, while the rich predominantly lives in low density areas. When training on Toronto data, the deep learning model learns to associate visual features associated with high-density with low income.  As a result, when predicting using London data – the same patterns emerge. The high-density city core as predicted as being low income and low-density outer city as predicted as higher income. Yet London's highest income groups live in the city center at high density areas. Similar patterns emerge when transferring from Los Angeles and New York. It is, however, visible from the prediction map from New York that the city's center with tall buildings in London are correctly predicted as higher income neighborhoods with a belt-like circle around the center. This is in line with what we would expect as a substantial part of the high-income population in New York lives in the very densely populated Manhattan area. We do not observe this pattern in Los Angeles where the city is known for its sprawl and most of its rich living in low density suburbs. Los Angeles predicts somewhat better than New York for poor areas outside the city center.

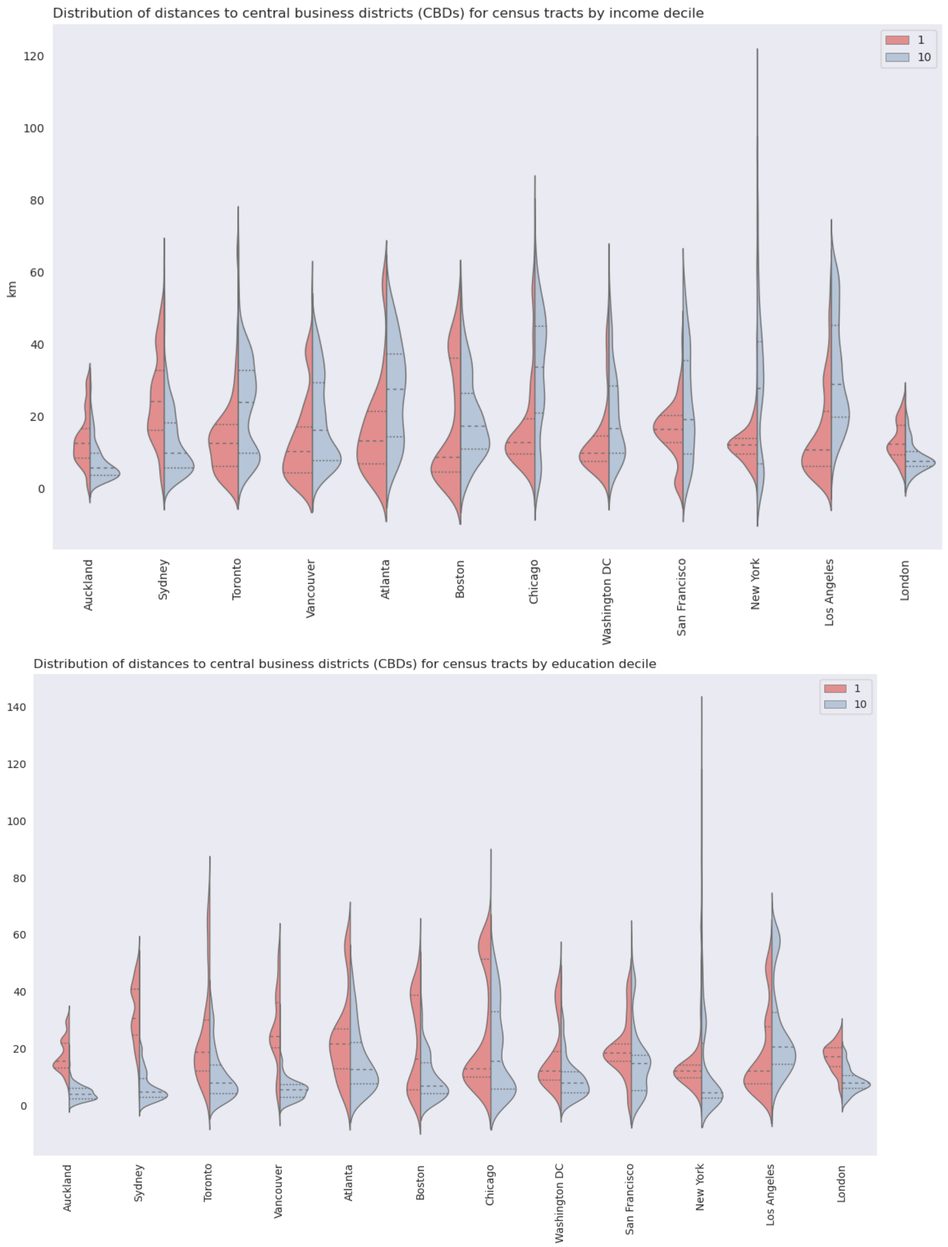


**Figure S5.** Comparison of distributions of distances to central business districts (CBDs) for census tracts for the best-off 10% (decile 1 in blue) and worst-off 10% (decile 10 in red).

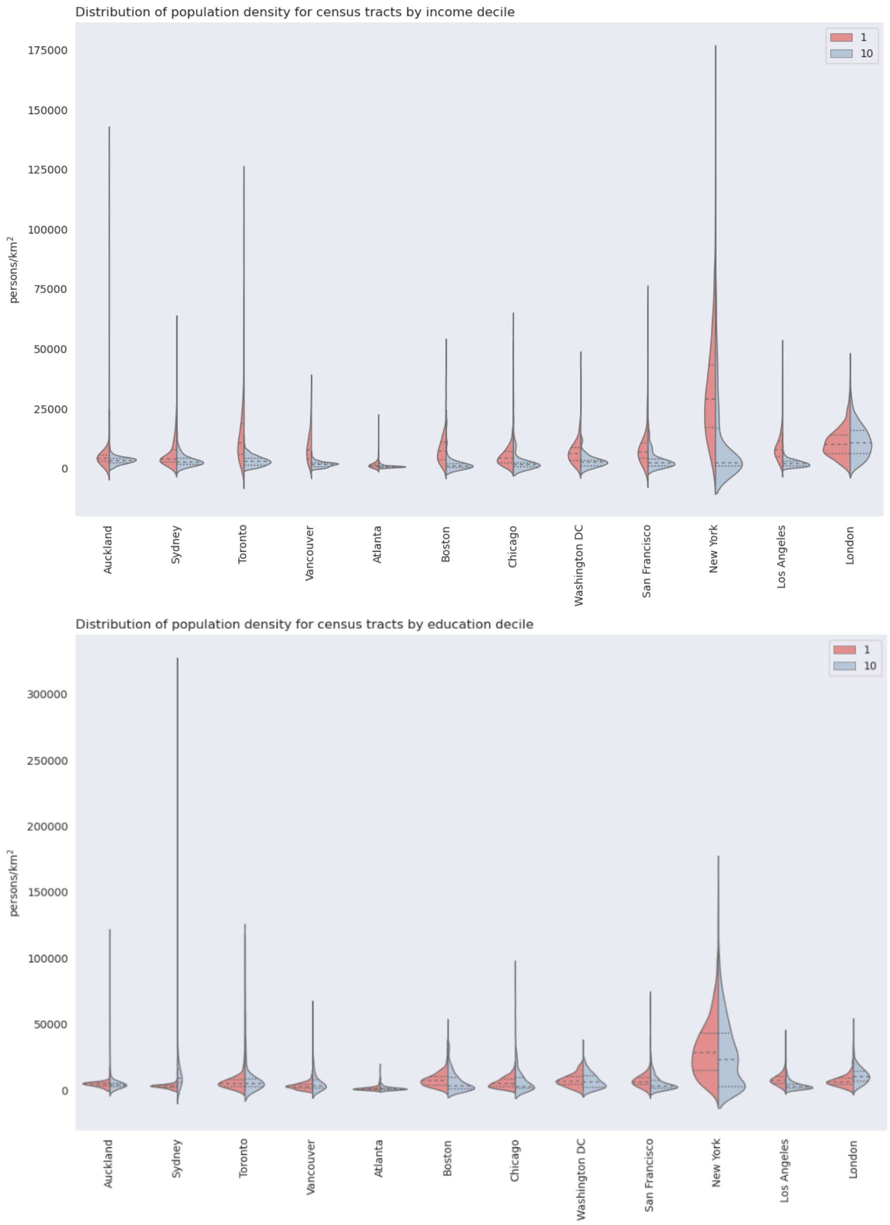


**Figure S6.** Comparison of distributions of population density for census tracts for the best-off 10% (blue) and worst-off 10% (red).

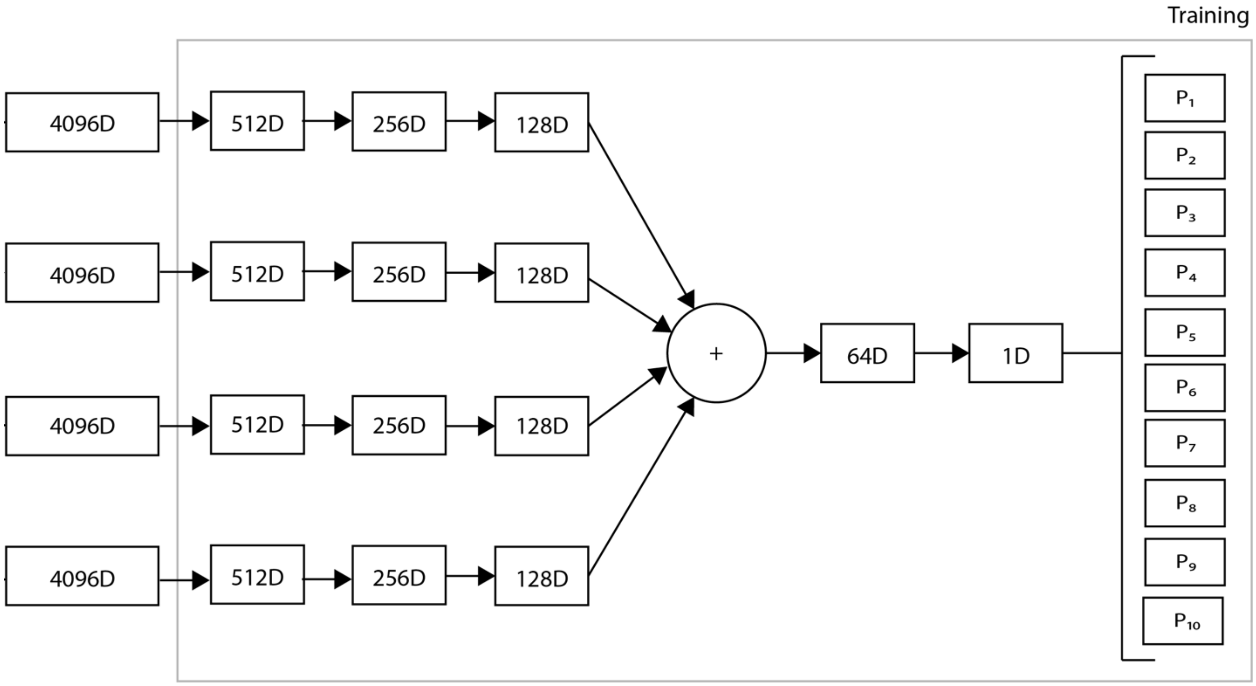


**Figure S7. The architecture of the network.** The network used all four 4096D extracted features from each location jointly, which were then aggregated and fed into the final layer to yield a single 1D continuous value between 0 and 1 using the sigmoid function. This single value was then used to compute the probabilities for each of the 10 decile classes as an ordinal classification task.

**Table S1.** Characteristics of cities included in our analysis.

| City name | City population (millions) | Number of census tracts (CTs) | Average census tract population | Number of images per census tract | Total number of images | Number of CTs with images |
|---|---|---|---|---|---|---|
| **Sydney** | 5.2 | 9,870 | 400 | 20 | 758,720 | 9,484 |
| **Auckland** | 1.3 | 7,932 | 168 | 20 | 607,520 | 7,594 |
| **Toronto** | 5.9 | 7,525 | 787 | 20 | 596,080 | 7,451 |
| **Vancouver** | 2.6 | 3,445 | 714 | 20 | 274,080 | 3,426 |
| **Atlanta** | 4.5 | 2,014 | 2,161 | 30 | 241,440 | 2,012 |
| **Boston** | 4.1 | 2,922 | 1,375 | 30 | 350,400 | 2,970 |
| **Chicago** | 8.6 | 5,499 | 1,395 | 30 | 659,280 | 5,494 |
| **Los Angeles** | 12.1 | 7,724 | 1,605 | 30 | 926,400 | 7,720 |
| **New York** | 18.3 | 8,990 | 1,362 | 30 | 1,071,720 | 8,931 |
| **San Francisco** | 3.2 | 2,206 | 1,521 | 30 | 264,600 | 2,205 |
| **Washington** | 4.5 | 2,806 | 1,689 | 30 | 334,920 | 2,791 |
| **London** | 8.2 | 4,835 | 1,690 | 30 | 579,240 | 4,827 |

**Table S2. Measurement performances for all city pairs.** Mean absolute errors (MAE) achieved when predicting worst-off and best-off deciles by (A) income and (B) educational attainment. Higher values of MAE suggest poorer performances.

## (A) Income

| **Poorest income decile** | | | | | | | | | | | | |
|---|---|---|---|---|---|---|---|---|---|---|---|---|
| | Auckland | Sydney | Toronto | Vancouver | Atlanta | Boston | Chicago | DC | San Francisco | Los Angeles | New York | London |
| Auckland | 1.79 | 2.62 | 2.70 | 2.76 | 3.71 | 3.04 | 4.89 | 3.64 | 3.20 | 3.79 | 2.13 | 2.54 |
| Sydney | 2.82 | 1.64 | 3.29 | 3.18 | 3.19 | 2.02 | 4.09 | 2.32 | 1.88 | 3.08 | 2.68 | 2.20 |
| Toronto | 3.43 | 3.42 | 1.16 | 1.66 | 2.36 | 1.61 | 2.37 | 2.11 | 2.29 | 2.03 | 1.60 | 3.10 |
| Vancouver | 3.41 | 3.66 | 1.48 | 1.39 | 3.21 | 1.65 | 2.33 | 2.17 | 2.90 | 1.81 | 1.90 | 3.02 |
| Atlanta | 3.71 | 3.20 | 4.60 | 4.49 | 1.46 | 3.11 | 3.84 | 2.35 | 3.27 | 3.66 | 4.49 | 3.72 |
| Boston | 3.55 | 2.93 | 2.17 | 2.55 | 3.17 | 1.20 | 1.90 | 2.18 | 2.09 | 1.91 | 1.98 | 3.23 |
| Chicago | 3.96 | 3.11 | 2.68 | 3.92 | 2.20 | 2.35 | 1.06 | 1.40 | 2.81 | 2.06 | 3.29 | 2.99 |
| Washington DC | 4.06 | 3.44 | 2.69 | 2.20 | 2.13 | 1.70 | 1.85 | 1.02 | 2.57 | 3.71 | 3.05 | 2.11 |
| San Francisco | 3.24 | 3.15 | 2.30 | 2.50 | 2.53 | 1.56 | 2.61 | 1.85 | 1.14 | 1.64 | 2.43 | 2.70 |
| Los Angeles | 2.94 | 3.01 | 1.85 | 2.44 | 2.81 | 1.60 | 2.24 | 1.92 | 1.66 | 1.15 | 2.05 | 2.71 |
| New York | 3.59 | 3.84 | 1.84 | 1.78 | 3.13 | 1.48 | 2.01 | 2.41 | 3.12 | 1.70 | 0.93 | 3.01 |
| London | 2.86 | 2.67 | 2.22 | 2.82 | 3.11 | 1.82 | 2.88 | 1.76 | 2.68 | 2.40 | 3.08 | 1.23 |

| **Wealthiest income decile** | | | | | | | | | | | | |
|---|---|---|---|---|---|---|---|---|---|---|---|---|
| City name | Auckland | Sydney | Toronto | Vancouver | Atlanta | Boston | Chicago | DC | San Francisco | Los Angeles | New York | London |
| Auckland | 1.15 | 1.87 | 2.72 | 2.59 | 2.35 | 3.36 | 3.72 | 2.23 | 2.47 | 2.34 | 4.10 | 3.71 |
| Sydney | 1.87 | 1.13 | 2.28 | 1.75 | 2.24 | 1.54 | 2.43 | 2.10 | 1.69 | 1.65 | 2.27 | 1.85 |
| Toronto | 4.51 | 3.25 | 1.27 | 1.35 | 1.92 | 1.77 | 2.16 | 1.74 | 1.92 | 1.82 | 2.99 | 4.85 |
| Vancouver | 5.01 | 3.62 | 2.80 | 1.06 | 2.48 | 2.12 | 2.71 | 2.26 | 2.52 | 2.05 | 2.32 | 4.41 |
| Atlanta | 3.55 | 2.76 | 2.52 | 2.16 | 1.05 | 2.28 | 2.05 | 2.06 | 2.14 | 2.01 | 2.84 | 4.40 |
| Boston | 4.28 | 2.52 | 1.84 | 1.36 | 2.38 | 1.11 | 1.91 | 2.20 | 1.84 | 1.62 | 1.67 | 2.41 |
| Chicago | 4.36 | 3.18 | 2.31 | 1.94 | 1.43 | 1.48 | 1.02 | 1.96 | 1.62 | 1.49 | 1.77 | 1.75 |
| Washington DC | 3.69 | 3.29 | 2.13 | 1.33 | 1.77 | 1.70 | 1.49 | 1.33 | 1.75 | 1.86 | 2.19 | 2.03 |
| San Francisco | 4.57 | 3.17 | 2.93 | 1.47 | 1.96 | 1.95 | 2.45 | 2.11 | 1.15 | 1.38 | 2.58 | 2.26 |
| Los Angeles | 3.83 | 2.94 | 2.08 | 1.33 | 2.18 | 1.80 | 2.09 | 2.01 | 1.61 | 0.95 | 2.84 | 3.96 |
| New York | 4.90 | 3.98 | 3.03 | 1.71 | 2.92 | 1.68 | 2.52 | 2.54 | 2.70 | 2.20 | 1.01 | 4.41 |
| London | 3.00 | 2.07 | 2.05 | 1.60 | 2.32 | 1.47 | 2.29 | 2.18 | 1.55 | 1.75 | 2.07 | 0.79 |

## (B) Educational attainment

| Lowest educational attainment decile | | | | | | | | | | | | |
|---|---|---|---|---|---|---|---|---|---|---|---|---|
| | Auckland | Sydney | Toronto | Vancouver | Atlanta | Boston | Chicago | DC | San Francisco | Los Angeles | New York | London |
| Auckland | 1.12 | 1.67 | 3.60 | 2.33 | 3.59 | 5.95 | 5.43 | 4.15 | 3.08 | 3.95 | 5.27 | 1.81 |
| Sydney | 1.94 | 1.11 | 3.28 | 2.22 | 3.38 | 4.83 | 5.08 | 3.05 | 2.17 | 3.03 | 5.49 | 1.56 |
| Toronto | 2.95 | 2.55 | 1.62 | 2.79 | 3.20 | 3.21 | 4.41 | 2.60 | 2.20 | 2.91 | 5.14 | 1.89 |
| Vancouver | 2.92 | 2.21 | 2.51 | 1.47 | 2.94 | 2.90 | 3.04 | 1.96 | 1.71 | 1.87 | 5.68 | 1.81 |
| Atlanta | 2.76 | 2.08 | 3.07 | 2.65 | 2.15 | 4.59 | 4.43 | 2.73 | 2.65 | 3.07 | 5.44 | 1.62 |
| Boston | 3.41 | 2.39 | 2.84 | 2.94 | 3.09 | 0.82 | 3.41 | 2.58 | 1.99 | 2.38 | 4.78 | 1.99 |
| Chicago | 3.55 | 2.89 | 2.51 | 2.83 | 2.36 | 2.74 | 1.31 | 1.46 | 1.92 | 1.60 | 5.30 | 1.69 |
| Washington DC | 2.87 | 2.34 | 2.98 | 2.48 | 2.67 | 2.47 | 3.13 | 1.07 | 1.70 | 3.03 | 5.18 | 1.60 |
| San Francisco | 3.15 | 2.36 | 3.06 | 2.80 | 2.89 | 2.86 | 4.04 | 2.45 | 0.88 | 1.62 | 4.45 | 1.65 |
| Los Angeles | 2.97 | 3.14 | 2.68 | 2.47 | 2.79 | 2.14 | 2.26 | 1.77 | 1.14 | 0.81 | 2.63 | 2.53 |
| New York | 3.83 | 4.56 | 3.19 | 3.71 | 3.01 | 1.02 | 2.53 | 2.31 | 2.31 | 1.35 | 1.32 | 5.07 |
| London | 2.61 | 2.11 | 2.89 | 2.58 | 3.11 | 3.79 | 4.29 | 2.95 | 2.52 | 3.11 | 5.16 | 0.89 |

| **Highest educational attainment decile** | | | | | | | | | | | | |
|---|---|---|---|---|---|---|---|---|---|---|---|---|
| | Auckland | Sydney | Toronto | Vancouver | Atlanta | Boston | Chicago | DC | San Francisco | Los Angeles | New York | London |
| Auckland | 0.91 | 1.05 | 1.92 | 1.66 | 1.62 | 3.33 | 2.69 | 1.76 | 2.48 | 2.12 | 3.18 | 1.94 |
| Sydney | 1.65 | 0.61 | 1.64 | 1.26 | 1.75 | 1.95 | 2.01 | 1.62 | 1.84 | 1.37 | 1.71 | 1.64 |
| Toronto | 2.63 | 1.41 | 0.75 | 0.92 | 1.68 | 1.26 | 1.61 | 1.55 | 1.70 | 1.89 | 1.39 | 1.73 |
| Vancouver | 3.94 | 1.55 | 1.46 | 0.68 | 1.97 | 1.22 | 1.25 | 1.51 | 1.66 | 1.48 | 1.40 | 1.37 |
| Atlanta | 2.18 | 1.24 | 1.50 | 1.34 | 0.83 | 1.86 | 1.71 | 1.62 | 1.95 | 1.76 | 1.79 | 1.58 |
| Boston | 3.47 | 1.36 | 1.33 | 1.14 | 2.24 | 1.04 | 1.41 | 1.74 | 1.68 | 1.67 | 1.26 | 1.53 |
| Chicago | 4.47 | 1.68 | 1.83 | 1.87 | 1.49 | 1.36 | 0.70 | 1.70 | 2.26 | 1.47 | 1.20 | 1.30 |
| Washington DC | 2.81 | 1.20 | 1.61 | 1.16 | 1.51 | 1.50 | 1.11 | 1.09 | 1.40 | 1.57 | 1.26 | 1.19 |
| San Francisco | 3.85 | 1.50 | 1.42 | 1.28 | 1.61 | 1.89 | 1.70 | 1.67 | 1.00 | 1.31 | 3.08 | 1.64 |
| Los Angeles | 3.70 | 3.88 | 2.01 | 1.73 | 2.86 | 2.84 | 1.97 | 3.20 | 1.71 | 0.83 | 3.70 | 2.96 |
| New York | 5.73 | 5.96 | 3.67 | 2.59 | 3.08 | 3.18 | 2.97 | 4.18 | 3.36 | 2.19 | 0.64 | 4.47 |
| London | 2.07 | 1.04 | 1.40 | 1.43 | 1.55 | 1.61 | 1.60 | 1.80 | 1.71 | 1.55 | 1.57 | 0.79 |
